# Screening and Characteristics of Marine *Bacillus velezensis* Z-1 Protease and Its Application of Enzymatic Hydrolysis of Mussels to Prepare Antioxidant Active Substances

**DOI:** 10.3390/molecules27196570

**Published:** 2022-10-04

**Authors:** Jing Lu, Yu Zhao, Rong Hu, Yu Cheng, Junhuan Qin, Jie Yang, Yaowei Fang, Mingsheng Lyu, Shujun Wang

**Affiliations:** 1 Jiangsu Key Laboratory of Marine Bioresources and Environment, Jiangsu Ocean University, Lianyungang 222005, China; 2 College of Food Science and Engineering, Jiangsu Ocean University, Lianyungang 222005, China

**Keywords:** protease, *Bacillus velezensis*, mussel, enzymatic hydrolysis, antioxidation

## Abstract

*Bacillus velezensis* is a type of microorganism that is beneficial to humans and animals. In this work, a protease-producing *B. velezensis* strain Z-1 was screened from sludge in the sea area near Qingdao (deposit number CGMCC No. 25059). The response surface methodology was used to analyze protease production, and the optimal temperature was 37.09 °C and pH 7.73 with the addition of 0.42% NaCl, resulting in maximum protease production of 17.64 U/mL. The optimum reaction temperature and pH of the protease of strain Z-1 were 60 °C and 9.0, respectively. The protease had good temperature and pH stability, and good stability in solvents such as methanol, ethanol and Tween 80. Ammonium, NH_4_^+^,and Mn^2+^ significantly promoted enzyme activity, while Zn^2+^ significantly inhibited the enzyme activity. The protease produced by strain Z-1 was used for the enzymolysis of mussel meat. The mussel hydrolysate exhibited good antioxidant function, with a DPPH free radical removal rate of 75.3%, a hydroxyl free radical removal rate of 75.9%, and a superoxide anion removal rate of 84.4%. This study provides a reference for the application of *B. velez* protease and the diverse processing applications of mussel meat.

## 1. Introduction

The free radicals produced by the metabolic process in the human body are highly oxidizing and destructive, and accelerate oxidative aging of the body, causing a series of cardiovascular diseases, immune system dysfunction, and even cancer [1]. Antioxidant substances play a role in protecting the body and delaying aging by inhibiting the formation of free radicals or by providing hydrogen electrons to remove free radicals [2]. They are widely used for the treatment of cardiovascular diseases [3], traumatic brain injury [4], Alzheimer’s disease [5] and other diseases. Long-term studies have revealed that compared to terrestrial organisms, marine organisms have more abundant antioxidant substances, complete functions and novel structures, which are of significant development and research value [6].

Mussels belong to the mollusk phylum, and are bivalve mollusks [7], also known as mussels and mussels, which are economically important shellfish worldwide [8]. Mussels are rich in various proteins and fatty acids, including eight essential amino acids and fatty acids required by the human body [9]. Owing to the insufficient development of mussel processing technology, most mussels in the market are traded in the form of fresh sales for direct sale and the added value of the product is low [10]. Microbial enzymatic hydrolysis of mussel meat to get active substances has been confirmed to be highly stable and effective, which has mild conditions, can improve the properties of proteins and enhance various activities [11]. In comparison with commercial enzymes, it has the advantages of low cost and high efficiency and can be hydrolyzed to obtain more abundant peptide segments, amino acids, and polysaccharides [12]. Several studies have revealed that mussel hydrolysates have anti-aging, anti-oxidant [13,14], anti-cancer [15], antibacterial [16,17], anti-inflammatory [18], and blood pressure reduction properties [19].

*Bacillus velezensis* is an endogenous probiotic widely used for biological control [20]. *B. velezensis* is characterized by easy separation and culture, a broad antibacterial spectrum [21], stable antibacterial effect [22], and high biological safety [23]. In addition, *B. velezensis* has various glycose and protease activities such as cellulase [24], amylase [25], tannase [26], xylanase [27], and protease activities [28,29], and thus has wide application prospects in food processing.

At present, there are only a few applied studies on protease production by *B. velezensis*. To fully develop marine resources and promote the rational utilization of mussel resources, a strain of *B. velezensis* Z-1 producing protease was screened from marine sludge. The protease produced by strain Z-1 was used to hydrolyze mussel meat and study the antioxidant activity of the hydrolysate. This is a hot research topic and a functional factor with development prospects. Simultaneously, it improved the comprehensive application value of mussels and was feasible and practical.

## 2. Results and Discussion

### 2.1. Strain Screen and Identification

#### 2.1.1. Screening of Strains

The 10 strains with larger clear circles obtained by primary screening were inoculated on the fermentation enzyme-producing medium and fermented on a shaking table at 37 °C and 180 rpm for 24 h. The fermentation broth was then centrifuged to obtain the supernatant, and the protease activity of each strain was measured according to the Folin method to obtain the strain with the highest protease activity, which was named Z-1.

#### 2.1.2. Morphological Biological Identification of Strain Z-1

**Figure 1:**
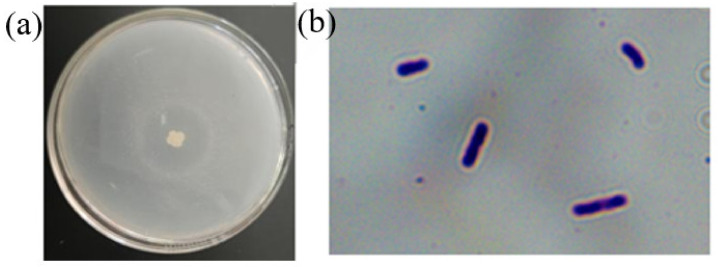
Colony morphology of strain Z-1 (**a**); Gram stain diagram of strain Z-1 (**b**).

The Z-1 colony was white, slightly yellow, and larger in size. The colony edge was irregular, and the surface was rough with wrinkles. In addition, there was a clear transparent ring around the colony, as shown in Figure 1, with similar colony-type morphological characteristics. Strain Z-1 was rod-shaped, observed under a microscope oil lens, and stained purple, indicating that it was a gram-positive bacterium according to the Gram staining kit instructions.

#### 2.1.3. Physiological and Biochemical Characteristics of Strain Z-1

The color changes in each reagent tube were observed, and their characteristics were determined according to the operating instructions of the physiological and biochemical reagent tubes. As summarized in Table 1, strain Z-1 has several basic characteristics of *B. velezensis*, such as methyl red, V-P, nitrate reduction, H_2_S gas generation, and urease. It can grow under conditions of 7% NaCl and pH 5.7, producing acid and no gas. It can use various sugars to secrete urease and amylase, which is consistent with the experimental results of Qiu [30]. Based on the morphological characteristics, physiological and biochemical reaction characteristics of the colonies, and the identification manual of common bacterial systems and Berger’s bacteria, strain Z-1 was preliminarily identified as *B. velezensis.*

#### 2.1.4. Molecular Biological Identification of Strain Z-1

**Figure 2:**
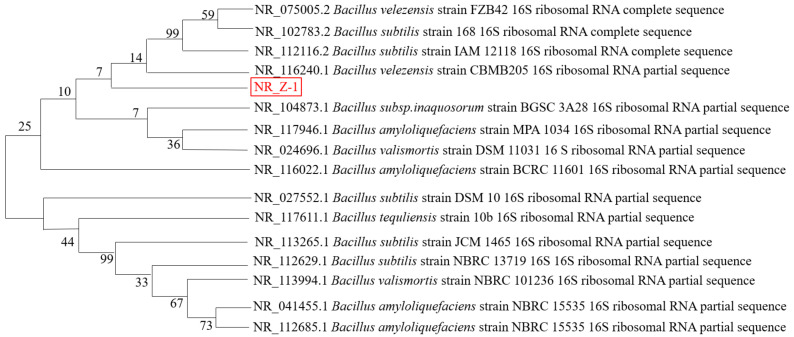
Phylogenetic tree of strain Z-1 constructed based on 16S rRNA.

The 16S rRNA gene sequence of the sequenced strain Z-1 was submitted to the GenBank database, where the homology of BLAST results with those of *Bacillus velezensis* was 99.8%. Gene sequences with close genetic relationships were selected for multiple comparisons using MEGA, and the neighbor-joining method was used to construct a phylogenetic tree, as shown in Figure 2. From the phylogenetic tree, strain Z-1 was the most similar to *B. velezensis.*, and it was identified as *Bacillus velezensis* by combining colony morphological characteristics, Gram staining, physiological and biochemical identification, and phylogenetic tree analysis.

### 2.2. Optimization Analysis of Protease Production by Fermentation of Strain Z-1

#### 2.2.1. Analysis of Single Factor Experiment Results

**Figure 3:**
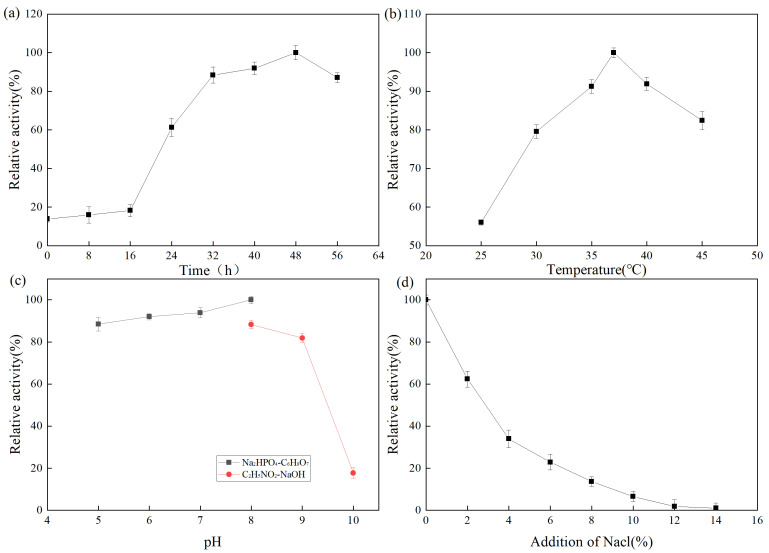
(**a**) Protease production curve by fermentation of strain Z-1; (**b**)Effect of temperature on enzyme production by fermentation of strain; (**c**) Effect of pH on enzyme production by fermentation of strain; (**d**) Effect of NaCl addition on enzyme production by fermentation of strain.

The fermentation enzyme production of the strain was expressed as relative enzyme activity. A higher relative enzyme activity indicates higher enzyme production. The effect of fermentation time on protease production by Z-1 is shown in Figure 3. From 0 to 16 h, the number of strains was small and the enzyme production rate was slow. At 16–48 h, the strain exhibited active metabolism and synthesized various metabolites, enzymes, and coenzymes. After 48 h, the medium was nutrient-deficient, a large number of toxic metabolites accumulated, the pH value decreased, some strains began to die, and the enzyme production rate rapidly decreased. The effect of fermentation temperature on protease production by strain Z-1 is shown in Figure 3b. The results demonstrated that the strain could produce protease at 25–45 °C, and the enzyme production rate increased with the increase in temperature from 25–37 °C, and then decreased gradually at 37 °C. The optimal enzyme production temperature was 37 °C. The effect of the fermentation pH on protease production by strain Z-1 is shown in Figure 3c. Strain Z-1 could ferment and produce enzymes at pH 5–10, but it accelerated when the pH was 5–8, and slowed when pH > 8. Protease production by strain Z-1 was higher under neutral and weakly alkaline conditions, with an optimal pH of 8. With the increase in NaCl addition, the enzyme yield of strain Z-1 decreased, indicating that NaCl inhibited protease production by the strain.

#### 2.2.2. Analysis of Results of Enzyme Production Optimized by Response Surface Methodology

To improve protease production by strain Z-1, we first performed single-factor experiments. Using maltose as a carbon source and peanut meal as a nitrogen source could effectively improve the protease secretion of strain Z-1 (Figure S1). Based on the single factor experimental results, according to the principle of central composite design, and using Design Expert 8.0.6 software, the temperature, pH and NaCl addition were selected as the influencing factors and regarded as the independent variables A, B and C. The levels of each variable were represented by −1, 0, and 1, and the independent variables were coded, as summarized in Table S1, and Box-Behnken experiment was designed with protease activity (R1) as the response value. The experimental design and results are summarized in Table S2.

(1) Model establishment and variance analysis

The experimental data of protease activity (R1) were fitted by quadratic multiple regression using Design Expert software, and the quadratic polynomial regression model of protease activity (R1) with temperature (A), pH (B), and NaCl addition (C) was obtained as follows:

R1 = 16.26 − 0.07A + 1.3B − 4.27C + 0.12AB − 0.23AC−1.15BC − 2.85A^2^ − 5.8B^2^ − 3.74C^2^

Design Expert software was used to analyze the variance and significance of the quadratic model. The results are summarized in Table S3. The *p*-value of the model was less than 0.01, and the analysis of variance of the model was significant. P_A_ > 0.05 indicated that temperature had no significant effect on the enzyme production of strain Z-1 within the temperature range of 35–39 °C; P_B_ < 0.05, indicated that pH had a significant effect on the enzyme production of strain Z-1 within the pH range of 6–9, P_C_ < 0.01 indicated that the addition of 0–2% NaCl had an extremely significant effect on the enzyme production of strain Z-1, and the influencing factor was C > B > A. The missing value *p*-value = 0.0653 > 0.05 was not significant, determination coefficient R^2^ = 0.9796, and adjustment coefficient Radj^2^ = 0.9534. The numerical value was close to 1, indicating that the regression equation had a good fit with the experiment, and that it could be used to analyze and predict the optimal enzyme production conditions. In the formula, the coefficient of the quadratic term is negative and the parabola of the model is downward, indicating the existence of a maximum point. In the primary term of the R1 regression model, B is a significant factor; C is an extremely significant factor; and A^2^, B^2^, and C^2^ in the secondary term are the extremely significant factors of the model.

(2) Response surface analysis

**Figure 4:**
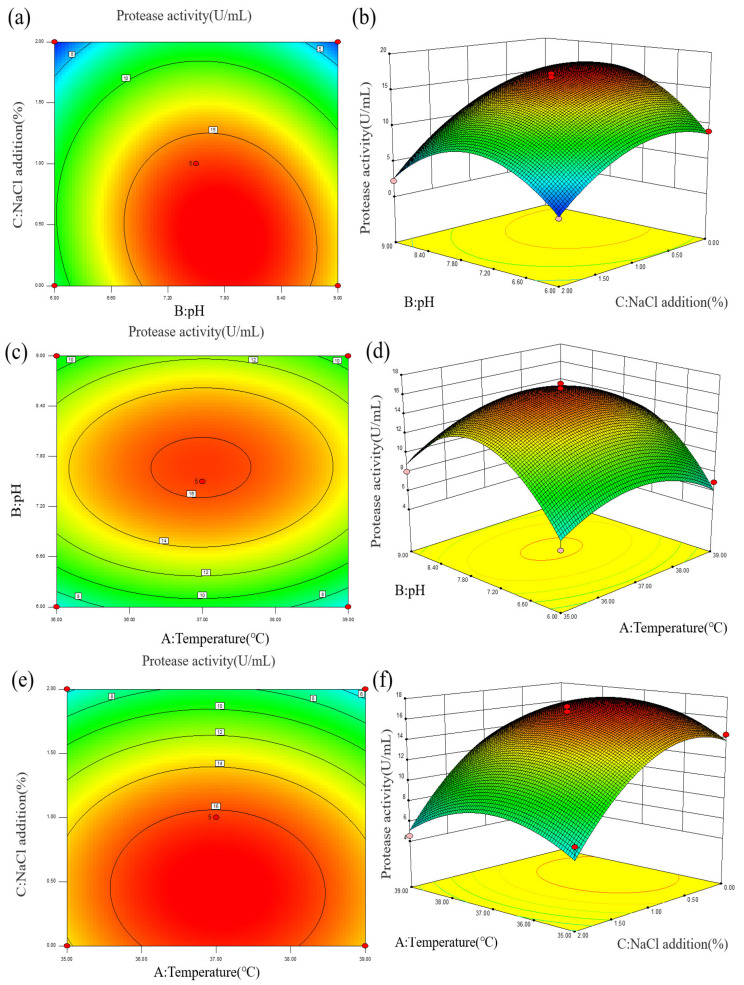
(**a**,**b**) Effects of fermentation pH and NaCl addition on protease activity; (**c**,**d**) Effects of fermentation pH and temperature on protease activity; (**e**,**f**) Effects of fermentation NaCl addition and temperature on protease activity.

To analyze the effects of various parameters on enzyme activity more intuitively, a contour line and response surface diagram of the interaction of factors on enzyme activity was constructed, as shown in Figure 4 and Table S3. The curves for AB, AC, and BC in the response surface diagram were relatively gentle, and the linearity of AB, BC, and AC in the contour diagram tended to be circular, indicating that the interaction of the three factors had no significant effect on enzyme activity. Both the addition of 0–2% NaCl and the pH range of 6.0–9.0 had significant effects on the protease production of strain Z-1. The total protease activity was predicted to be the highest at a fermentation temperature of 37.09 °C, pH of 7.73, and 0.42% NaCl addition, which was the optimal enzyme production condition as shown in Table S4.

(3) Verification experiment

To test the accuracy of the response surface methodology, 37.09 °C, pH 7.73, 0.42% NaCl addition and protease activity 17.64 U/mL were selected according to Table S4 for the validation experiment. Combined with the actual equipment conditions, 37.10 °C, pH 7.73, and 0.42%NaCl addition were finally selected as the experimental conditions for five parallel experiments. The actual enzyme activity was measured as 16.92 U/mL, and the error value was approximately 5%. The response surface model was proven to be reliable.

### 2.3. Enzymatic Properties of Strain Z-1 Protease

#### 2.3.1. Effects of Temperature on Protease Activity of Strain Z-1

**Figure 5:**
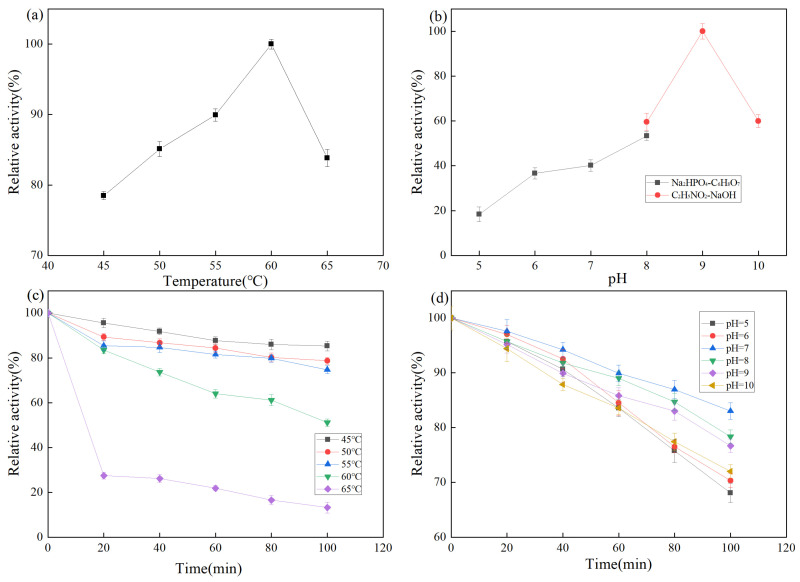
(**a**) Effect of temperature on enzyme activity; (**b**) Effect of pH on enzyme activity; (**c**) Effect of temperature on the stability of enzyme activity; (**d**) Effect of pH on the stability of enzyme activity.

Temperature is an important factor affecting enzyme activity. As shown in Figure 5a, proteases exhibit a relative enzyme activity of more than 75% at 45–65 °C, with the highest activity at 60 °C. A protease activity of more than 80% is maintained at 65 °C, which is higher than that of common proteases such as neutral protease (45–50 °C), alkaline protease (40–55 °C), and papain (50–60 °C). The temperature stability of the protease from strain Z-1 is shown in Figure 5c. The results demonstrated that the protease was stable within 100 min at 45–55 °C and maintained 80% enzyme activity. At the optimal reaction temperature of 60 °C for 1 h, more than 60% of enzyme activity was maintained. When the temperature was above 65 °C, the stability of the enzyme became poor and the enzyme activity was rapidly lost, which was not conducive to the use of the enzyme.

#### 2.3.2. Effects of pH on Protease Activity of Strain Z-1

The pH affects the dissociation of relevant active groups at the active site of the enzyme molecule, thus affecting the binding of the enzyme to the substrate. The effect of pH on protease activity of strain Z-1 is shown in Figure 5b. Relative enzyme activity was lower at pH 5–7, higher at pH 8–10, and the optimal reaction pH was 9. The results demonstrated that the enzyme activity was higher under alkaline conditions. The pH stability of the protease of strain Z-1 is shown in Figure 5d, and the relative enzyme activity was higher in the pH 7–9 range, whereas the relative enzyme activity was lower under the conditions of pH > 10 and pH < 7. It could maintain 80% activity after reacting the optimal pH of 9 for 100 min, indicating that the enzyme reacted for a long time in an environment with relatively high acidity and alkalinity with rapid enzyme activity loss, which was suitable for long-time reactions under weak alkaline conditions [31]. Combined with these conclusions, it is a basic protease.

#### 2.3.3. Effects of Metal Ions on Protease Activity of Strain Z-1

As summarized in Table 2, at metal ion concentrations of 5 and 25 mM, different metal ions had different effects on the protease activity of strain Z-1. As summarized in Table 2, Ca^2+^, Mg^2+^, Ba^2+^, NH_4_^+^, Li^+^, and Mn^2+^ activated proteases to a certain extent. The addition of Mn^2+^ increased enzyme activity by 1.47 times at most while K^+^, Na^+^, Zn^2+^, Ni^2+^, and Sr^2+^ inhibited protease activity. However, Zn^2+^ exhibited significant inhibition, retaining only 5.94% of enzyme activity. Ca^2+^ and Mn^2+^ maintained the native conformation of the protease by limiting its folding, thereby enhancing its stability and proteolytic activity [32], whereas other metal ions altered the initial structure of the enzyme, resulting in a decrease in enzyme activity [33]. The concentration of metal ions had a greater effect on protease activity. When the concentrations of Ca^2+^, Mg^2+^, Ba^2+^, NH_4_^+^, Li^+^, and Mn^2+^ increased from 5 to 25 mM, the effect of Ca^2+^ on protease changed from promotion to inhibition while those of Mg^2+^, Ba^2+^, NH_4_^+^, Li^+^, and Mn^2+^ changed from inhibition to promotion.

#### 2.3.4. Effects of Reducing Agent, Denaturant, Organic Solvent and Other Compounds on Protease Activity

As summarized in Table 3, the enzyme exhibited good stability in the presence of various reagents. The reducing agents GSH, β-mercaptoethanol, and cysteine do not promote protease activity, but retain most of the activity. Several denaturants and protease inhibitors can inhibit enzymes. The enzyme activity was generally reduced in the presence of the surfactant [32], retaining 77.83% of the enzyme activity in the presence of TritonX-100 and an increase of 19.9% in Tween 80. In organic solvents, ethyl acetate strongly inhibited enzyme activity while 10% final concentrations of methanol, dimethyl sulfoxide, and ethanol promoted enzyme activity. With an increase in ethanol concentration, ethanol changed from the promotion to inhibition of enzyme activity, and the inhibition effect was gradually evident.

#### 2.3.5. Zymography of *B. velezensis* Z-1 extracellular proteases

Among these several substrates, Casein is the most suitable substrate for this enzyme (Figure S2a). In order to analyze the types of bacterial extracellular proteases, a serine protease inhibitor (PMSF) and a metalloprotease inhibitor (O-Phenanthrone) were used in combination with the effect of protease inhibitors on enzyme activity, and electrophoresis experiments on enzyme inhibitor substrate soaking activity were carried out. As shown in Figure S2b, the extracellular protease spectrum of *Bacillus velezensis* showed a number of enzymatic bands. After being soaked in PMSF, the enzymatic bands at 25–70 kDa were significantly reduced; after O-Phenanthroline treatment, one enzymatic hydrolysis band was reduced at 70–100 kDa. Combined with the inhibition rate of PMSF on protease activity of about 50% in Table 3, the extracellular protease of *Bacillus velezensis* Z-1 was mainly serine protease.

### 2.4. Oxidation Resistance Analysis of Mussel Enzymolysis Product Y1

#### 2.4.1. Effect of Hydrolysis Time on Antioxidant Activity of Mussels

**Figure 6:**
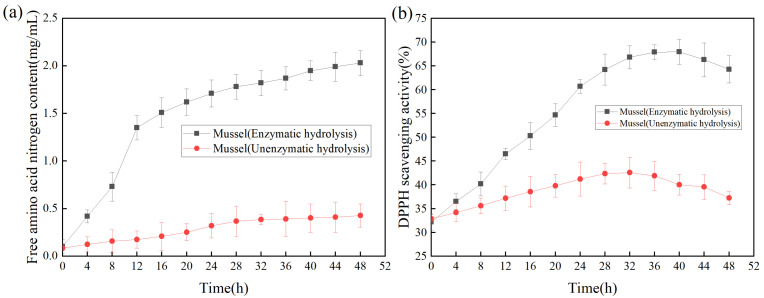
(**a**) Effect of enzymolysis time on free amino acid nitrogen; (**b**)Effect of enzymolysis time on DPPH scavenging activity.

The productivity curve could monitor the change of product yield under specific conditions to improve product yield and reduce the cost of production [34]. As shown in Figure 6a, with the increase of reaction time, the content of free amino acid nitrogen in the hydrolysate was significantly higher than that without the addition of enzyme, indicating that the addition of protease made the mussel more fully hydrolyzed. As shown in Figure 6b, the DPPH scavenging rate was used as the index of antioxidant activity to determine the effect of enzymolysis time on the antioxidant activity of the products [35]. Within 0–8 h, the antioxidant activities of the products subjected to enzymolysis and non-enzymolysis were similar; The antioxidant activity of the products was the highest at 40 h, while the DPPH scavenging rate was gradually decreased after 40 h, suggesting that long-time enzymolysis might lead to the degradation or inactivation of relevant antioxidant proteins, thereby reducing the antioxidant activity of the products [36].

#### 2.4.2. Analysis of DPPH Radical Scavenging Activity of Enzymolysis Products

**Figure 7:**
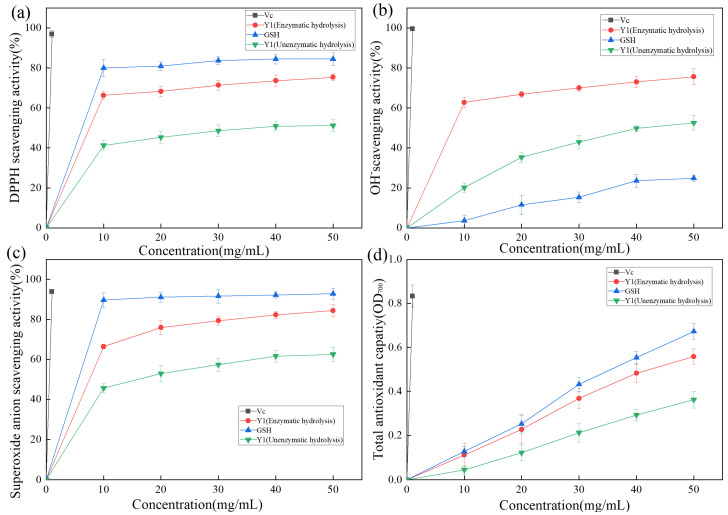
(**a**) Scavenging activity of Y1 DPPH radical of enzymolysis product; (**b**) Scavenging activity of Y1 hydroxyl radical of enzymolysis product; (**c**) Superoxide anion radical scavenging activity of enzymolysis product; (**d**) Determination of the reducing force of enzymolysis product Y1.

DPPH contains free radicals. Owing to its stable nature and low cost, it is often used to evaluate the antioxidant activity of various substances in food [37]. The hydrogen supply capacity of the sample to be tested determines the DPPH scavenging effect. As shown in Figure 7a, DPPH radical clearance increased with increasing sample concentration. Vc exhibited the highest DPPH radical clearance, reaching 96.9% at a concentration of 1 mg/mL. GSH was confirmed to be a reducing peptide [38] with a good antioxidant function [39,40]. After enzymatic hydrolysis, the DPPH free radical clearance rate of product Y1 was significantly increased, which increased about 1.5 times. When the sample Y1 (Enzymatic hydrolysis) concentration was 50 mg/mL, the DPPH free radical clearance rate of product Y1 reached 75.3%, which was 10% lower than that of GSH, indicating that Y1 had a good DPPH free radical clearance effect.

#### 2.4.3. Analysis of Hydroxyl Radical Scavenging Activity of Enzymolysis Products

The hydroxyl radical has an oxidation potential of 2.80 eV, it exhibits extremely strong oxidation ability, and the bioactive peptide exhibits a strong removal effect on the hydroxyl radical [41]. As shown in Figure 7b, Vc exhibited the strongest hydroxyl radical-scavenging activity, with a clearance of 99.2% at 1 mg/mL. The hydroxyl radical scavenging activity of Y1 was stronger than that of GSH. When the sample Y1 (Enzymatic hydrolysis) concentration was 50 mg/mL, the hydroxyl radical scavenging rate of Y1 was 75.9%, which was three times that of GSH.

#### 2.4.4. Analysis of Superoxide Anion Radical Scavenging Activity of Enzymolysis Products

The free superoxide anion is generated based on the metabolic process of organisms, which causes macromolecular structures, such as proteins, lipids, and nucleic acids, to be crosslinked or broken in the body to lose their original structure and active functions, accelerates the aging of the body, causes biological damage, and induces diseases [42]. As shown in Figure 7c, Vc exhibited the highest superoxide anion radical clearance, reaching 93.9% at a concentration of 1 mg/mL. Product Y1 (Enzymatic hydrolysis) exhibited good superoxide anion radical activity, with a clearance rate of 84.4% at a concentration of 50 mg/mL.

#### 2.4.5. Reduction Force Analysis of Enzymolysis Products

Antioxidants reduce ferric ions to divalent ions. The reaction between iron oxide and iron chloride generates Prussian blue. Based on the principle of Prussian blue, which has a maximum absorption peak at OD_700_, and the measured OD_700_ representing the reducing force, the relationship between the concentration of zymolyte Y1 and the total reducing force was studied, as shown in Figure 7d. The total reducing force increased significantly with increasing sample concentration. When the sample Y1 (Enzymatic hydrolysis) concentration was 50 mg/mL, it was approximately equal to the reducing force of 41 mg of GSH.

#### 2.4.6. Comprehensive Application Analysis of Antioxidant Activity of Mussel Enzymolysis Products

Wang [43] isolated a novel antioxidant peptide (BNH-P7) from blue mussel hydrolysate, and its amino acid sequence was identified as Tyr-Pro-Pro-Ala-Lys (YPPAK), which exhibited good scavenging activity against DPPH free radicals, hydroxyl free radicals, and superoxide anion free radicals. Rajapakse [13] isolated a heptapeptide sequence, HFGBPFH, from *Mytilus ocellatus*, which scavenges superoxide (98%), hydroxyl radicals (96%), carbon centers (91%), and DPPH radicals (72%) at 200 μg/mL with IC values of 21, 34, 52, and 96 μM, respectively. In brief, the experimental data and analytical literature revealed that the enzymatic products of mussels have good antioxidant activity and broad application prospects in the preparation of food-derived antioxidants.

## 3. Materials and Methods

### 3.1. Samples and Medium

Reagents: casein, yeast powder, sucrose, maltose, glucose, tapioca, tryptone, fish meal peptone, peanut meal, bran, sodium chloride, folin, Na_2_CO_3_, trichloroacetic acid, NaOH, HCl, ethanol, glutathione, ascorbic acid, etc. (Sinopharm Chemical Reagent Co., Ltd., Shanghai, China).

Sample A2 was obtained from sludge in the sea area near Qingdao (Shangdong, China), collected in a sealed bag, marked with time and date, and stored in a refrigerator at 4 °C for later use.

LB medium: yeast powder 5 g/L, tryptone 10 g/L, sodium chloride 30 g/L; casein culture medium: yeast powder 10 g/L, casein 10 g/L, agar 20 g/L, prepared with aged seawater (filtering), pH 7.0; seed culture medium: yeast powder 5 g/L, tryptone 10 g/L, aged seawater, pH 7.0; and fermentation medium: yeast powder 10 g/L, casein 10 g/L, aged seawater, pH 7.0.

A pH meter (Shanghai Yiheng Scientific Instrument Co., Ltd., Shanghai, China), high-speed refrigerated centrifuge (Ebend China Co., Ltd., Shanghai, China), vacuum freeze dryer (Shanghai Bajiu Industrial Co., Ltd., Shanghai, China), thermostatic water bath (Shanghai Yiheng Scientific, Shanghai, China), and a full-wavelength microplate reader (Thermo Fisher, New York, NY, USA) were employed.

### 3.2. Screening of Protease Producing Strains

Screening: One gram of activated sludge A2 sample was weighed, diluted to 10^0^, 10^−1^, 10^−2^, 10^−3^, 10^−4^, 10^−5^, and 10^−6^ with a sterile water gradient, 200 μL was sucked on the screening medium with a pipette, coated evenly with a coating rod, and then placed in a constant temperature incubator at 37 °C for 24 h. A strain with a larger transparent circle was selected for isolation, purification, and preservation according to the size of the transparent circle produced by the strain.

Re-screening: Several strains obtained from the primary screening were cultured to the second seed solution, inoculated into the fermentation medium for enzyme production with 10% inoculation amount, cultured in a shaking table at 37 °C and 180 rpm for 24 h, centrifuged to obtain supernatant, and the protease activity was determined by the Flynn method. The strain with the highest enzyme activity was named Z-1, and the next step was to study strain Z-1.

### 3.3. Determination of Protease Activity

Refer to Liu [44] for the determination of protease activity with appropriate modifications. One milliliter of protease solution and 1 mL of 2% casein solution (boric acid buffer, pH 9) were mixed in a water bath at 50 °C for 10 min, followed by the addition of Folin’s reagent (0.5 mL), and the absorbance was determined at 680 nm. Protease activity per unit was defined as the number of enzymes that digest 1 μg of tyrosine per minute.

### 3.4. Biomorphological Identification of B. velezensis Z-1

Colony characteristics: LB medium inoculated with the strain was cultured at 37 °C for 18 h, followed by casein medium with the inoculation loop, and then cultured at 37 °C for 24–36 h to observe the morphological characteristics of the strain.

Gram staining: The operations were performed according to the instructions of the Gram staining kit (Beijing Suleibao Technology Co., Ltd., Beijing, China), including smear, primary staining, mordant staining, decolorization, counterstaining, and other steps. Gram staining of the strain was performed using an oil microscope.

Physiological and biochemical experiments: The appropriate types of physiological and biochemical reagent tubes were selected (Hangzhou Binhe Microbial Reagent Co., Ltd., Hangzhou, China), and 100 μL of seed liquid was sucked into each reagent tube using a pipette and cultured for 24 h in a 37 °C incubator. The color changes of the reagent tubes were observed, and positive and negative results were determined according to the manufacturer’s instructions.

For molecular biological identification: The genome of strain Z-1 was extracted according to the operation of bacterial the DNA extraction kit (Tiangen Biochemical Technology Co., Ltd., Beijing, China) and amplified by PCR. The 16S rRNA sequences of the sequenced bacteria were compared with the homology of the GenBank database, 15 strains with the closest homology were selected, and the phylogenetic tree of strain Z-1 was drawn using MEGA 4.0 software (Mega Limited, Auckland, New Zealand).

### 3.5. Optimization of Protease Production by Fermentation of B. velezensis Z-1

The bacterial liquid was cultured in secondary seed liquid, and the inoculated amount was 10% of the fermentation enzyme production medium. The medium was cultured under different temperatures, pH, time, NaCl addition, initial carbon source, and nitrogen source conditions (the same as those previously mentioned) in a single factor experiment. The fermentation liquid was centrifuged to obtain the supernatant, and the relative enzyme activity under different fermentation conditions was measured to study the enzyme production characteristics of strain fermentation.

On the basis of single factor experiment and in reference to the Amany A. Hassabo response surface methodology [45], Box-Behnken was designed using Design expert 8.0.6 software (Borregard Industries, Minneapolis, MN, USA) with temperature, pH, and NaCl addition as variables, and the test results were analyzed.

### 3.6. Characterization of Enzymatic Properties of B. velezensis Protease

Effect of temperature and pH on enzyme activity: The enzyme liquid obtained by fermentation was placed at different temperatures (45, 50, 55, 60, and 65 °C) or pH values (5, 6, 7, 8, 9, and 10), and the protease activity was determined using the flint method. The effects of temperature and pH on the protease activity were also compared.

Temperature and pH stability study: The enzyme liquid was placed at different temperatures (45, 50, 55, 60, and 65 °C) or pH values (5, 6, 7, 8, 9, and 10) for 20, 40, 60, 80, and 100 min, respectively. The relative enzyme activities under each condition were measured, and the temperature and pH stabilities were compared.

Effect of metal ions on enzyme activity: In accordance with the final metal ion concentrations of 5 and 25 mM, Ca^2+^, K^+^, Na^+^, Zn^2+^, Mg^2+^, Ni^2+^, Ba^2+^, Sr^2+^, NH_4_^+^, Li^+^ and Mn^2+^ were selected to prepare a metal ion solution, and the effect of metal ions at the two concentrations on the enzyme activity was determined.

Effect of compounds such as reducing agents, denaturants, and organic solvents on enzyme activity: According to the method of ZHU [46] and making appropriate improvements, the reducing agents glutathione (GSH), β-mercaptoethanol, cysteine, denaturant SDS, urea, protease inhibitors PMSF, EDTA, surfactant TritionX-100, and Tween 80, and the organic solvents methanol, ethanol, dimethyl sulfoxide, and ethyl acetate were selected and formulated into appropriate concentrations to determine the effect of each compound on enzyme activity.

Zymography of B.velezensis Z-1 extracellular proteases: Casein, gelatin, bovine serum albumin and skim milk were selected to determine the substrate specificity of strain Z-1. According to the method of enzyme spectrum and appropriated modifications [47], the samples containing protease were incubated with non-reducing Laemmli protein buffer (2.5% SDS) in an ice bath for 1 h. Native-PAGE was prepared, and 0.1% casein was added. Electrophoresis was carried out in Tris-Gly (SDS-free) buffer. After the completion of electrophoresis, the gel was washed with 2.5% Triton X-100 for 20 min, which was repeated twice to remove SDS and restore protease activity, and then washed with ultrapure water twice. The gel was cut into three parts, one part was immersed in C_2_H_5_NO_2_-NaOH (pH 9.0) buffer, and the other two parts were soaked in a C_2_H_5_NO_2_-NaOH (pH 9.0) buffer containing PMSF and O-Phenanthreone with a final concentration of 2 mM, respectively. The samples were incubated for 1 h at 37 °C, followed by washing, staining and decolorization.

### 3.7. Preparation and Separation of Mussel Enzymolysis Products by B. velezensis Protease

#### 3.7.1. Determination of Free Amino Acid Nitrogen

The effect of enzymolysis time on the degree of hydrolysis was explored with free amino acid nitrogen as the index to determine the degree of hydrolysis, and the content of amino acid nitrogen was determined by the double-indicator formaldehyde titration method [48]. The reaction solution was composed of 2 mL sample solution with different enzymatic hydrolysis times, 5 mL neutral formaldehyde solution, 5 mL distilled water, two drops 0.05% bromothymol blue and four drops 0.5% phenolphthalein alcohol solution, and was titrating with 0.05 M sodium hydroxide standard solution. The volume of sodium hydroxide consumed by the blank titration and the volume of sodium hydroxide consumed by the sample titration were recorded, and the free amino acid nitrogen content was calculated according to the following formula.

$$N=\frac{\left(V1-V2\right)\times C\times 0.014}{W}$$

*N*: Content of amino acid nitrogen in the sample, mg/mL; *V*1: Consumption of sodium hydroxide volume in the titration sample solution, M; *V*2: Consumption of sodium hydroxide volume in the blank control test, M; 0.014: The mass of nitrogen equivalent to 1.00 g sodium hydroxide standard titration solution; *C*: Actual concentration of sodium hydroxide standard solution used in titration, M; *W*: Quality of sample, g.

#### 3.7.2. Effect of Enzymolysis Time on Antioxidant Activity

Referring to the view of Nikhita [35], the effect of enzymolysis time on antioxidant activity was studied with DPPH scavenging rate as an index to determine the highest productivity. The reaction solution was composed of 0.1 mL sample solution with different enzymatic hydrolysis times and 0.1 mL DPPH (0.1 mM, prepared in ethanol solution), and the absorbance at 517 nm was measured after being placed in a dark environment for 30 min [49]. The unezymolytic mussel sample was used as a blank control.

$$\mathrm{DPPH}\;\mathrm{radical}\;\mathrm{scavenging}\;\mathrm{rate}\left(\%\right)=\left(1-\frac{A-B}{C}\right)\times 100\%$$

*A*: Absorbance of mixture of DPPH solution and enzymolysis product; *B*: Absorbance of enzymolysis product; *C*: Absorbance of mixture of DPPH solution and water.

#### 3.7.3. Preparation and Separation of Antioxidants

Dry mussel powder (20 g) was dissolved in 400 mL pure water, mixed with 1 g *B. velezensis* protease, and enzymolyzed on a shaking table at 40 °C and 180 rpm for 40 h. The enzymolysis liquid was removed and centrifuged to obtain the supernatant, which was filtered to remove suspended matter, and then ultrafiltered with 8 and 5 kDa molecular filter membranes to obtain three-grade products with different molecular sizes. Fraction Y1 was separated with a molecular weight less than 5 kDa and freeze-dried at −80 °C for later use.

### 3.8. Evaluation of Antioxidant Activity of Enzymolysis Products by B. velezensis Protease

The lyophilized powder of Y-1 enzymolysis product was prepared in an aqueous solution at concentrations of 10, 20, 30, 40, and 50 mg/mL. The DPPH radical scavenging activity, superoxide anion radical scavenge activity, hydroxyl radical scavenge activity and determination of reducing power of the solution that was tested refer to previous articles [49].

### 3.9. Statistical Analysis

All experiments were performed three times in the same way, and all samples were analyzed in three parallels. All data results were expressed as mean ± standard deviation.

## 4. Conclusions

In this study, a strain of *Bacillus velezensis* was screened from the sludge in the sea area near Qingdao, and its entire genome has been confirmed to be highly safe. Using the protease secreted by the strain to treat mussel meat, the mussel enzymatic products exhibited good antioxidant activity, which has broad application prospects for the development of enzyme-producing applications of *Bacillus velezensis* and research on the high-value transformation of mussel meat.

## Figures and Tables

**Table 1:** Physiological and biochemical characteristics of strain Z-1.

Project	Result	Project	Result
Gram stain	+	Urea	+
7% NaCl salt tolerance	+	Glucose	+
PH 5.7 growth	+	Sucrose	+
V-P	+	Maltose	−
Gelatin liquefaction	+	Cellobiose	−
Amylase hydrolysis	+	Lactose	−
Nitrate reduction	+	Gum sugar	+
H2S gas production	−	Xylose	+
Methyl red	−	Aescin	+
Propionate	−	Salicin	−
Mannitol	+	Ornithine decarboxylase	−
Sorbitol Indole	+ −	Lysine decarboxylase	−

Note: “+” indicates positive; “−” is negative.

**Table 2:** Effect of metal ions on protease activity.

Metal Ion	Relative Enzyme Activity (%) (5 mM)	Relative Enzyme Activity (%) (25 mM)
Blank	100 ± 0.43	100 ± 0.64
Ca^2+^	102.44 ± 1.62	91.43 ± 2.72
K^+^	82.58 ± 1.32	93.30 ± 3.20
Na^+^	56.73 ± 2.63	99.39 ± 0.68
Zn^2+^	51.83 ± 1.02	5.94 ± 0.84
Mg^2+^	72.10 ± 0.26	106.45 ± 1.61
Ni^2+^	71.67 ± 2.89	43.63 ± 2.63
Ba^2+^	65.93 ± 2.36	104.47 ± 0.57
Sr^2+^	74.22 ± 1.28	98.70 ± 2.40
NH_4_^+^	71.88 ± 1.60	135.91 ± 1.39
Li^+^	69.86 ± 0.57	103.73 ± 2.38
Mn^2+^	89.12 ± 0.80	147.18 ± 0.93

**Table 3:** Effects of several chemical reagents on protease activity.

Reagent Type	Reagent Name	Final Concentration	Relative Enzyme Activity(%)
Control	Ultrapure water	-	100 ± 0.68
Reductant	Glutathione	10 mM	91.32 ± 1.58
	β-Mercaptoethanol	10 mM	89.41 ± 1.22
	Cysteine	10 mM	98.63 ± 2.12
Denaturant	SDS	0.10%	71.00 ± 0.48
	Urea	4 M	78.32 ± 1.65
Protease Inhibitor	EDTA	5 mM	79.27 ± 2.56
	PMSF	5 mM	52.75 ± 0.78
Surfactant	TritonX-100	0.10%	77.83 ± 1.50
	Tween80	0.10%	119.89 ± 2.61
Organic solvent	Methyl alcohol	10%	113.24 ± 1.55
	Ethyl acetate	10%	45.43 ± 1.48
	Dimethyl sulfoxide	10%	111.58 ± 2.87
	Ethanol	10%	104.31 ± 0.45
	Ethanol	20%	29.90 ± 1.23
	Ethanol	30%	21.33 ± 0.84

## Data Availability

The data presented in this study are available in the present article.

## Supplementary Materials

The following supporting information can be downloaded at: https://www.mdpi.com/article/10.3390/molecules27196570/s1, Figure S1: Effect of carbon source on enzyme production by fermentation of strain (a); Effect of nitrogen source on enzyme production by fermentation of strain (b); Table S1: Box-Behnken Test Factors and Levels.; Table S2: Box-Behnken Experimental Design and Results.; Table S3: Analysis of variance of protease activity regression model.
