# Supplementary material for: Screening and Characteristics of Marine Bacillus velezensis Z-1 Protease and Its Application of Enzymatic Hydrolysis of Mussels to Prepare Antioxidant Active Substances
Source: Molecules. 2022 Oct 4;27(19):6570. doi: 10.3390/molecules27196570 (PMC9572009; doi:10.3390/molecules27196570)
Supplement: Supplementary file 1 [file molecules-27-06570-s001.zip › molecules-1918682-supplementary.pdf]

Figure S1 Effect of carbon source on enzyme production by fermentation of strain (a); Effect of nitrogen source on enzyme production by fermentation of strain (b).

Table S1 Box-Behnken Test Factors and Levels.

Table S2 Box-Behnken Experimental Design and Results.

Table S3 Analysis of variance of protease activity regression model.

Table S4 Response surface methodology for protease activity estimation.

Figure S2 Effects of different substrates on protease activity(a); Detection of proteases and their types of *Bacillus velezensis* Z-1 by inhibitorgelatin immersing gel zymography (b).

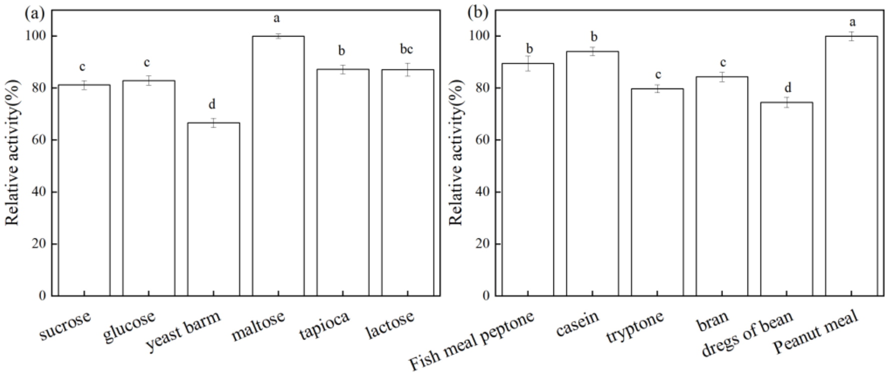

Figure S1 Effect of carbon source on enzyme production by fermentation of strain (a);

Effect of nitrogen source on enzyme production by fermentation of strain (b).

Table S1 Box-Behnken Test Factors and Levels.

Level	Temperature°C（A）	pH（B）	NaCl addition%（C）
-1	35	6	0
0	37	7.5	1
1	39	9	2

Table S2 Box-Behnken Experimental Design and Results.

Number	A	B	C	R1
1	35	6	1	5.54
2	39	6	1	6.94
3	35	9	1	8.06
4	39	9	1	9.93
5	35	7.5	0	14.36
6	39	7.5	0	12.90
7	35	7.5	2	6.90
8	39	7.5	2	4.53
9	37	6	0	8.93
10	37	9	0	13.67
11	37	6	2	2.07
12	37	9	2	2.22
13	37	7.5	1	17.15
14	37	7.5	1	15.64
15	37	7.5	1	16.21
16	37	7.5	1	16.66
17	37	7.5	1	15.66

Table S3 Analysis of variance of protease activity regression model.

Variance source	Sum of squares	Freedom	Mean square	F	P	Significance
Model	423.8	9	47.09	37.37	< 0.0001	Significant
A	0.039	1	0.039	0.031	0.865	-
B	13.53	1	13.53	10.73	0.0136	*
C	145.68	1	145.68	115.6	< 0.0001	**
AB	0.055	1	0.055	0.044	0.8401	
AC	0.21	1	0.21	0.16	0.6973	
BC	5.26	1	5.26	4.18	0.0803	
A^2	34.15	1	34.15	27.11	0.0012	
B^2	141.52	1	141.52	112.3	< 0.0001	
C^2	58.98	1	58.98	46.81	0.0002	
Residual	8.82	7	1.26	/	/	
Lack of Fit	7.12	3	2.37	5.57	0.0653	not significant
Pure Error	1.7	4	0.43	/	/	
Cor Total	432.62	16	/	/	/	

R^2=0.9796；$Radj^2$=0.9534

Table S4 Response surface methodology for protease activity estimation.

Number	Temperature (°C)	pH	NaCl addition (%)	Protease activity(U/mL)
1	37.09	7.73	0.42	17.6419
2	36.79	7.68	0.45	17.5882
3	37.28	7.70	0.46	17.5852
4	37.29	7.84	0.40	17.5848
5	37.20	7.71	0.51	17.5831
6	37.18	7.66	0.33	17.581
7	37.06	7.71	0.54	17.5737
8	37.05	7.80	0.54	17.57
9	37.30	7.86	0.38	17.5696
10	36.82	7.92	0.46	17.5299
11	37.26	7.79	0.25	17.5236
12	37.41	7.90	0.35	17.4886
13	37.03	7.60	0.25	17.4742
14	37.48	7.85	0.46	17.4593
15	36.73	7.98	0.37	17.4524

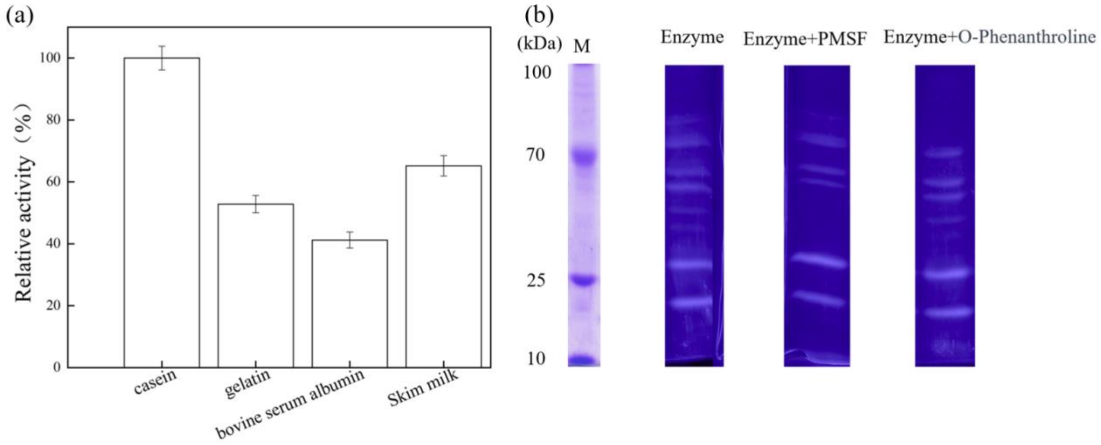

Figure S2 Effects of different substrates on protease activity(a); Detection of proteases and their types of *Bacillus velezensis* Z-1 by inhibitorgelatin immersing gel zymography (b).
